# Best–worst scaling methodology to evaluate constructs of the Consolidated Framework for Implementation Research: application to the implementation of pharmacogenetic testing for antidepressant therapy

**DOI:** 10.1186/s43058-022-00300-7

**Published:** 2022-05-14

**Authors:** Ramzi G. Salloum, Jeffrey R. Bishop, Amanda L. Elchynski, D. Max Smith, Elizabeth Rowe, Kathryn V. Blake, Nita A. Limdi, Christina L. Aquilante, Jill Bates, Amber L. Beitelshees, Amber Cipriani, Benjamin Q. Duong, Philip E. Empey, Christine M. Formea, J. Kevin Hicks, Pawel Mroz, David Oslin, Amy L. Pasternak, Natasha Petry, Laura B. Ramsey, Allyson Schlichte, Sandra M. Swain, Kristen M. Ward, Kristin Wiisanen, Todd C. Skaar, Sara L. Van Driest, Larisa H. Cavallari, Sony Tuteja

**Affiliations:** 1 University of Florida Clinical and Translational Science Institute, Gainesville, FL USA; 2 University of Florida College of Medicine, Gainesville, FL USA; 3 University of Minnesota Medical School, Minneapolis, MN USA; 4 University of Minnesota College of Pharmacy, Minneapolis, MN USA; 5 University of Florida College of Pharmacy, Gainesville, FL USA; 6 MedStar Health, Georgetown University Medical Center, Washington, DC USA; 7 Indiana University School of Medicine, Indianapolis, IN USA; 8 Nemours Children’s Health, Jacksonville, FL USA; 9 University of Alabama Heersink School of Medicine, Birmingham, AL USA; 10 School of Medicine and Pharmacy, University of Colorado, Aurora, CO USA; 11 Durham VA Healthcare System, Durham, NC USA; 12 University of Maryland School of Medicine, Baltimore, MD USA; 13 University of North Carolina Medical Center, Chapel Hill, NC USA; 14 Nemours Children’s Health, Wilmington, DE USA; 15 University of Pittsburgh School of Pharmacy, Pittsburgh, PA USA; 16 Intermountain Healthcare, Salt Lake City, UT USA; 17 Moffitt Cancer Center, Tampa, FL USA; 18 Corporal Michael J. Cresenz VA Medical Center, Philadelphia, PA USA; 19 University of Michigan College of Pharmacy, Ann Arbor, MI USA; 20 North Dakota State University/Sanford Health, Fargo, ND USA; 21 Cincinnati Children’s Hospital Medical Center, Cincinnati, OH USA; 22 Fairview Pharmacy Services, Minneapolis, MN USA; 23 Vanderbilt University Medical Center, Nashville, TN USA; 24 University of Pennsylvania Perelman School of Medicine, Smilow Center for Translational Research, 3400 Civic Center Boulevard, Bldg. 421 11th Floor, Room 143, Philadelphia, PA 19104-5158 USA

**Keywords:** Best–worst scaling, Pharmacogenetic testing, Consolidated Framework for Implementation Research

## Abstract

**Background:**

Despite the increased demand for pharmacogenetic (PGx) testing to guide antidepressant use, little is known about how to implement testing in clinical practice. Best–worst scaling (BWS) is a stated preferences technique for determining the relative importance of alternative scenarios and is increasingly being used as a healthcare assessment tool, with potential applications in implementation research. We conducted a BWS experiment to evaluate the relative importance of implementation factors for PGx testing to guide antidepressant use.

**Methods:**

We surveyed 17 healthcare organizations that either had implemented or were in the process of implementing PGx testing for antidepressants. The survey included a BWS experiment to evaluate the relative importance of Consolidated Framework for Implementation Research (CFIR) constructs from the perspective of implementing sites.

**Results:**

Participating sites varied on their PGx testing platform and methods for returning recommendations to providers and patients, but they were consistent in ranking several CFIR constructs as most important for implementation: patient needs/resources, leadership engagement, intervention knowledge/beliefs, evidence strength and quality, and identification of champions.

**Conclusions:**

This study demonstrates the feasibility of using choice experiments to systematically evaluate the relative importance of implementation determinants from the perspective of implementing organizations. BWS findings can inform other organizations interested in implementing PGx testing for mental health. Further, this study demonstrates the application of BWS to PGx, the findings of which may be used by other organizations to inform implementation of PGx testing for mental health disorders.

**Supplementary Information:**

The online version contains supplementary material available at 10.1186/s43058-022-00300-7.

Contributions to the literature

- Best–worst scaling is a quantitative technique for eliciting individual preferences for products or services. Here we describe its application for eliciting importance of factors for implementing pharmacogenetic testing for antidepressants from an organizational perspective.
- Organizations that were early adopters of implementing pharmacogenetics were consistent in their rankings of factors within the Consolidated Framework for Implementation Research (CFIR) constructs as most important for implementation success.
- These findings exemplify the application of the BWS methodology to quantitatively prioritize a large number of factors with cognitive simplicity.

## Background

Depression and anxiety disorders are common in the US, affecting approximately one in five people [1]. These conditions are frequently treated with antidepressants; however, identifying optimal treatment(s) for a given patient can be challenging when considering side-effects, patient comorbidities, clinical symptoms, concomitant medications, and prior treatment history [2]. Antidepressant medication trials can be a frustrating process that may take weeks while patients wait for the medications to elicit their full effects. Up to 42% of the variation in response to antidepressants may be explained by genetic factors [3] and there is a growing body of research supporting the clinical utility of pharmacogenetic (PGx) information to guide drug dosing or selection [4].

Pharmacogenetic testing, like other novel interventions, is subject to similar challenges of uptake and adoption into healthcare. Assessing internal and external organizational barriers to implementation is an essential step to facilitate the adoption of novel interventions. Implementation science with its robust methods, including defined and validated frameworks, strategies, and outcome measures, can be leveraged to accelerate PGx implementation as applied to mental health clinical practice [5]. With the growing body of evidence motivating providers and patients to seek PGx testing to guide the prescribing of medications for treating mental health conditions, successful approaches to implementation for antidepressants need to be identified. One dynamic approach for the appraisal of choices in health-related settings is the stated preferences approach. Stated preferences approaches are increasingly being applied to as an assessment tool when engaging implementation stakeholders [6]. We highlight herein the utility of applying a stated preferences approach to evaluate the relative importance of constructs in the Consolidated Framework for Implementation Research (CFIR), and how latent class analyses may extend and enhance the interpretation of findings by identifying latent class membership among respondents according to their organizational characteristics.

## Methods

### Setting

This study was conducted within the Implementing Genomics in Practice (IGNITE) network—a multidisciplinary consortium in the United States (US) focused on the development, implementation, and dissemination of methods that integrate genomic medicine into clinical care [7, 8]. Informed by the CFIR, IGNITE researchers previously identified constructs that were critical to the adoption of genomic medicine and PGx [9–11]. In this paper, we focused on examining latent classes to better identify the specific factors within the CFIR that are important for the implementation of PGx testing for antidepressant prescribing by organizations. The survey targeted funded and affiliate members of the IGNITE network that had either implemented or were planning to implement clinical genotyping to guide antidepressant prescribing.

### Study procedures

We developed an electronic survey to (1) measure institutional and practice environment characteristics and (2) to evaluate factors important for the implementation of PGx testing to guide antidepressant therapy using stated preferences [11] (Additional file 1). Specifically, the experiment was designed using the best–worst scaling (BWS) technique—a methodology for assessing priorities by asking respondents what they view as best and worst amongst a given set of factors [12]. Respondents were asked to provide a consensus on prioritization from their site rather than respond based on their own perspectives only. The online survey was administered between September and December 2020 to 17 sites in the IGNITE network that had responded to a previous survey about institutional characteristics and programmatic drivers [13] and completed by organizational representatives leading implementation efforts with input from mental health providers. Prior to its administration, the survey was pilot tested among members of the research team with experience in PGx implementation. Both the survey data collection and the BWS analysis were conducted using Lighthouse Studio (Version 9.9.2; Sawtooth Software, Provo, UT).

### Measures

We used BWS to identify which factors were most important for implementing PGx for antidepressants. The BWS exercise evaluated constructs from the CFIR because it is a widely applied, stakeholder-engaging framework that has informed implementation efforts across IGNITE [9, 10]. The CFIR includes 37 constructs organized into five major domains that influence the implementation of interventions: (1) outer setting, (2) inner setting, (3) characteristics of individuals, (4) intervention characteristics, and (5) process of implementation. The constructs for each of the CFIR domains comprised 5 independent BWS exercises. A copy of the survey is available in the supplementary material.

### Experimental design

**Fig. 1:**
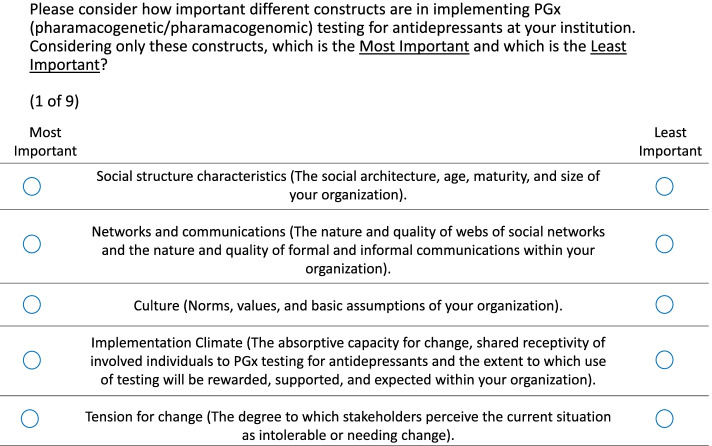
Example of one choice set from the best–worst scaling experiment representing the Inner Setting domain of the Consolidated Framework for Implementation Research

The BWS technique has been applied to evaluate preferences across a range of healthcare applications [12]. This technique is useful for quantitatively prioritizing a relatively large number of observed factors while maintaining cognitive and administrative simplicity [14]. BWS requires that respondents choose the best (highest ranking) and worst (lowest ranking) factors in a series of choice tasks, thus yielding the relative importance of these factors. The BWS exercise asked respondents to rank all constructs of the CFIR within each of its five domains—outer setting (4 factors evaluated in 3 choice sets), inner setting (14 factors evaluated in 9 choice sets), intervention characteristics (9 factors evaluated in 6 choice sets), characteristics of individuals (5 factors evaluated in 3 choice sets), and process (8 factors evaluated in 5 choice sets). In addition to CFIR constructs, the BWS domains included three additional constructs from the Genomic Medicine Integrative Research (GMIR) Framework [15] that may be relevant to PGx testing (social determinants of health, ability of system to educate patients and clinicians, and patient needs and resources: bio-psychosocial factors). The GMIR Framework was developed to inform the integration of genomic medicine into clinical practice and includes four domains: healthcare system factors, social determinants, clinician factors, and individual and family factors [15]. An example choice set is presented in Fig. 1.

### Data analysis

We used multinomial logistic regression in Sawtooth Software to estimate the probabilities of respondents choosing particular alternatives. Probability scores were then transformed into scaled probability scores to allow for comparisons across constructs. Additionally, we applied a Bayesian approach using a Monte Carlo Markov chain to compare and update respondents’ estimates on the basis of the distribution of preferences from other respondents. The individual utility estimates of each construct were averaged after 10,000 random draws.

We used Sawtooth Software’s latent class analysis module to identify latent classes of respondents with similar choice patterns. The latent class models assumed that the presentation of alternative constructs could have heterogenous effects on choices across latent classes of respondents. Given the small sample size in this study, we limited the latent class specification to two classes for each of the models. The latent class formula computes the probability that each participant is a member of each class. To account for this heterogeneity, the models assume that there are latent classes within the sample such that each class has preference weights that are identical within the class and that are systematically different from preference weights in other classes. Within each latent class, the preference weights were estimated using conditional logit. Summary statistics for site characteristics (whether PGx testing has been implemented or was being planned, and whether the participating organization is [or not] affiliated with an academic medical center) were estimated by latent class. The study was approved by the University of Florida Institutional Review Board. The Strengthening the Reporting of Observational Studies in Epidemiology (STROBE) statement: guidelines for reporting observational studies were used to guide research reporting (Additional file 2) [16].

## Results

### Relative importance scores for the overall sample

**Fig. 2:**
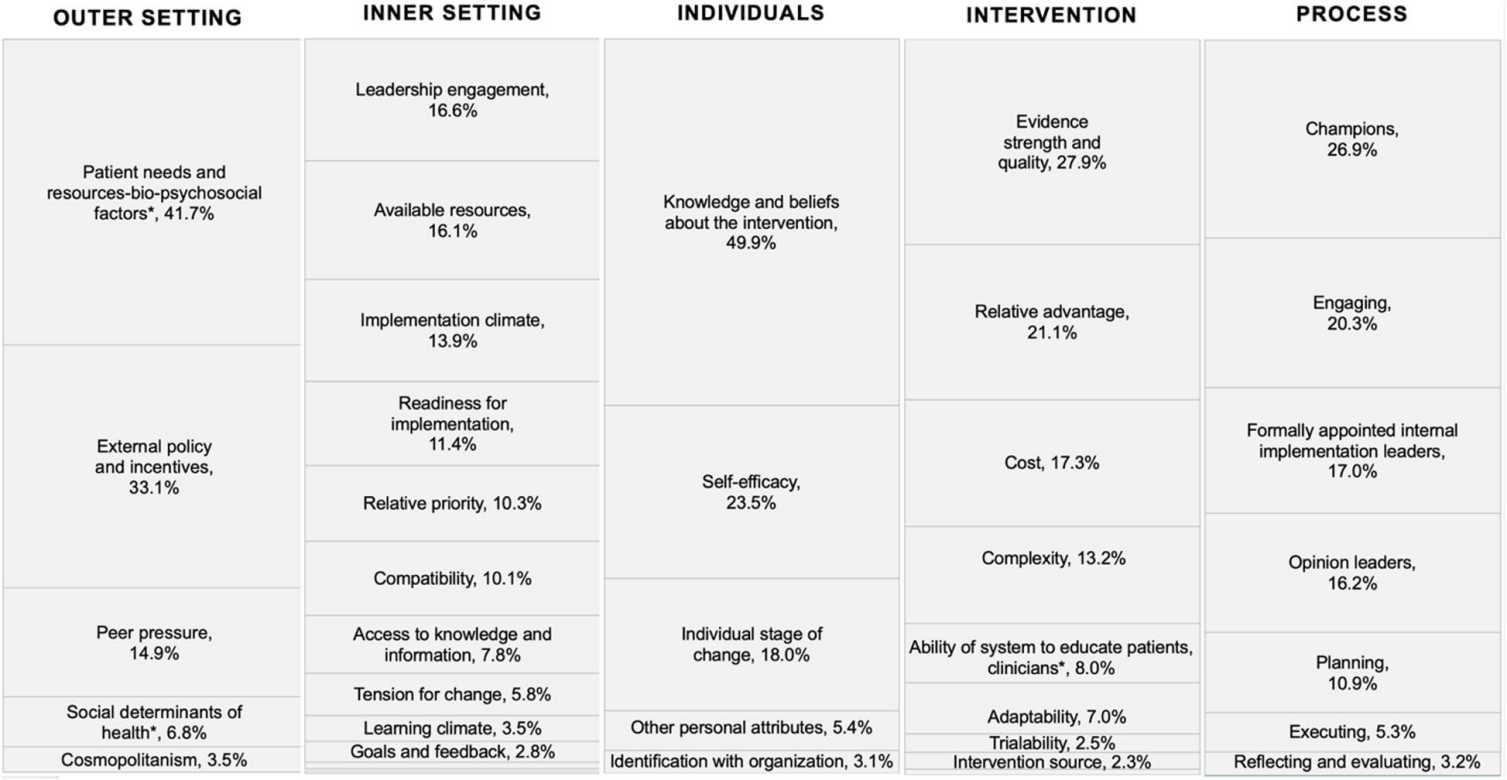
Relative importance of each construct from the Consolidated Framework for Implementation Research (CFIR) organized by domain applied to the implementation of pharmacogenetics testing to guide antidepressant treatment. *Additional constructs from the Genomic Medicine Integrative Research Framework

All 17 IGNITE sites that were invited to participate completed the survey (response rate = 100%) (Additional file 3). Results of the institutional and practice environment characteristics component of the survey have been reported elsewhere [11]. Figure 2 illustrates the relative importance scores for each construct organized by CFIR domain across the entire sample (*n* = 17). *Patient needs and resources—biopsychosocial factors* was rated as the most important construct (relative score, 41.7%) for *outer setting*, *leadership engagement* (relative score, 16.6%) for *inner setting*, *knowledge and beliefs about the intervention* (relative score 49.9%) for *characteristics of individuals*, *evidence strength and quality* (relative score, 27.9%) for *intervention characteristics*, and *champions* (relative score, 26.9%) for *process*.

### Relative importance scores and site characteristics by latent class

Table 1 summarizes the importance scores by latent class for the highest-ranked constructs within each CFIR domain, latent class membership (i.e., proportion of sites in each latent class), and site characteristics by class (i.e., proportion of sites within each class with the corresponding characteristic). For *outer setting*, the highest-ranked construct in class 1 (i.e., highest importance score) was *patient needs and resources—biopsychosocial factors* (42.7%) compared with *external policy and incentives* (49.5%) in class 2. For *inner* setting, the highest-ranked construct in class 1 was *available* resources (15.3%) compared with leadership engagement (19.6%) in class 2. For *characteristics of individuals*, the highest-ranked construct in class 1 was *individual stage of change* (40.8%) compared with *knowledge and beliefs about the intervention* (52.4%) in class 2. For *intervention characteristics*, the highest-ranked construct in class 1 was *relative advantage* (27.8%) compared with *evidence strength and quality* (29.0%) for class 2. Finally, for *process*, the highest-ranked construct in class 1 was *champions* (30.6%) compared with *formally appointed internal implementation leaders* (31.0%) in class 2. When compared with demographic characteristics of the organization, neither of the two classes was more likely than the other to have a particular identity (academic medical center or had already implemented PGx testing), although more centers that had already implemented were classified in class 2 and academic medical centers were more often classified in class 1.

**Table 1:** Importance scores and latent class membership for the highest-ranked constructs within each domain and site characteristics by class

Highest-ranked constructs within each domain	% (95% CI)	Latent class %	
		**1**	**2**
*Domain: Outer setting*			
1. Patient needs and resources-bio-psychosocial factors^a^	41.7 (38.1, 45.3)	42.7	38.6
2. External policy and incentives	33.1 (26.0, 40.2)	14.4	49.5
3. Peer pressure	14.9 (8.6, 21.3)	31.8	2.4
4. Social determinants of health^a^	6.8 (4.9, 8.7)	7.1	6.3
5. Cosmopolitanism	3.5 (0.9, 6.1)	4.0	3.1
Latent class membership		44.5	55.5
*Site characteristics by latent class membership*			
Implemented		85.7	70.0
Academic medical center		71.4	60.0
*Domain: Inner setting*			
1. Leadership engagement	16.6 (15.4, 17.7)	15.2	19.6
2. Available resources	16.1 (15.1, 17.1)	15.3	15.1
3. Implementation climate	13.9 (12.3, 15.6)	11.3	15.6
4. Readiness for implementation	11.4 (9.2, 13.7)	12.4	5.4
5. Relative priority	10.3 (7.4, 13.2)	8.3	13.9
Latent class membership		76.5	23.5
*Site characteristics by latent class membership*			
Implemented		69.2	100.0
Academic medical center		76.9	25.0
*Domain: Characteristics of individuals*			
1. Knowledge and beliefs about the intervention	49.9 (45.8, 54.0)	30.6	52.4
2. Self-efficacy	23.5 (18.4, 28.5)	4.9	25.3
3. Individual stage of change	18.0 (12.1, 23.9)	40.8	12.2
4. Other personal attributes	5.4 (2.7, 8.2)	3.1	7.8
5. Individual identification with organization	3.1 (0.2, 6.0)	20.5	2.2
Latent class membership		17.2	82.8
*Site characteristics by latent class membership*			
Implemented		66.7	78.6
Academic medical center		33.3	71.4
*Domain: Intervention characteristics*			
1. Evidence strength and quality	27.9 (25.9, 29.8)	24.0	29.0
2. Relative advantage	21.1 (17.0, 25.2)	27.8	16.3
3. Cost	17.3 (13.4, 21.1)	6.2	22.1
4. Complexity	13.2 (9.9, 16.6)	14.4	10.6
5. Ability of the healthcare system to educate individuals receiving care, families, clinicians ^a^	8.0 (5.0, 11.0)	2.6	12.4
Latent class membership		34.7	65.3
*Site characteristics by latent class membership*			
Implemented		83.3	72.7
Academic medical center		33.3	81.8
*Domain: Process*			
1. Champions	26.9 (23.2, 30.6)	30.6	25.9
2. Engaging	20.3 (16.1, 24.5)	29.5	10.0
3. Formally appointed internal implementation leaders	17.0 (11.5, 22.4)	3.0	31.0
4. Opinion leaders	16.2 (11.5, 21.0)	18.4	10.8
5. Planning	10.9 (8.2, 13.7)	15.7	8.5
Latent class membership		44.9	55.1
*Site characteristics by latent class membership*			
Implemented		62.5	88.9
Academic medical center		75.0	55.6

^a^Constructs from the Genomic Medicine Integrative Research (GMIR) Framework

## Discussion

This study demonstrates the use of BWS methodology to assess the prioritization of contextual factors for the implementation of PGx testing to guide antidepressant therapy. Our analyses revealed that patient needs and resources, leadership engagement, knowledge and beliefs about the intervention, evidence strength and quality, and identifying champions were the most important constructs across the five CFIR domains from the organizational perspective when implementing PGx testing. These preferences differed little by key characteristics of the organization (i.e., whether the site had implemented PGx testing and whether it was an academic medical center). Although BWS methods have been used to inform implementation of evidence-based practices [6], many applications focus on the patient or the individual receiving the intervention, and few studies have used stated preferences to assess the perspective of the implementing organization. The results from our study provide important information for institutions seeking to advance precision medicine approaches to mental health care, and the latent class analysis allows for identification of classes with different priorities.

Applying latent class analysis to the CFIR construct prioritization can inform the developing and tailoring of PGx testing and implementation strategies by identifying priorities by classes of implementers. There are several caveats of latent class analysis that merit further consideration. While grouping based on latent class facilitates data presentation and interpretation, participating organizations do not actually belong to a single group, and class membership for each organization is assigned based on the highest probability of belonging to one of the latent classes. Ranking priorities can help set a roadmap for planning implementation and the order in which certain implementation strategies should be deployed. The BWS rankings of the CFIR constructs were very similar by class membership with subtle differences. CFIR domains for which the rankings were different between the two classes included *Inner Setting*, where *available resources* was ranked higher in class 1 and *leadership engagement* was ranked higher in class 2; *Intervention characteristics*, where *relative advantage* was ranked higher in class 1 and *evidence strength and quality* was ranked higher in class 2; C*haracteristics of individuals*, *individual stage of change* was ranked higher in class 1 and *knowledge and beliefs about the intervention* in class 2; and *Process*, where preparing *champions* ranked higher in class 1 and *formally appointed internal implementation leader* ranked higher in class 2. These differences may reflect the stage of implementation as those centers that had already implemented PGx testing more often comprised membership in class 2.

Implementers and mental health clinicians highly value the strength and quality of the evidence linking genotype with drug response when designing their implementations [17, 18]. Such evidence informs decisions made in the implementation process, including which genes/alleles to test, which drugs and patient populations to target, and what clinical recommendations to provide based on genotype results. A challenge with evidence in the area of antidepressant pharmacogenetics is that while, several randomized controlled trials have shown the benefit of PGx testing [19–22] on remission rates in patients treated for depression, these trials used different combinations of genes and different proprietary dosing algorithms in tailoring antidepressants, which makes the interpretation of the clinical utility of PGx testing challenging for practicing clinicians. As such, implementers may need to review the entirety of the clinical evidence, and especially consider the strength of the evidence for genetic associations with antidepressant response, in designing implementations. Stakeholders are also weighing the advantage of PGx testing as compared to prescribing medications without testing, recognizing that these advantages need to be established in their specific clinical environment. [17]. Institutions who are more experienced with PGx implementation may recognize the value of leadership engagement in supporting the initial infrastructure needs to start PGx testing, as well as to help sustain the program with financial, capital, and personnel needs. For example, for genotyping to be performed during a hospitalization, the cost is covered under the diagnosis related group (DRG), but there must be leadership buy-in for this to happen and to be sustained as institutional priorities change. Institutions still in the planning phase are likely prioritizing an evaluation of the additional resources required to implement a PGx program, such as for hiring PGx experts, establishing and validating an in-house assay for performing the testing, and designing and building informatics resources to integrate PGx results into electronic health records [23]. Implementers placed emphasis on users’ knowledge and beliefs as the skilled and enthusiastic use of PGx testing will inform prescribing decisions and help optimize drug therapy for a patient. Preparing of physician champions is well-recognized strategy for implementation as champions can serve as resources for building clinical decision support and for educating and supporting other physicians in how to order testing and apply the results to prescribing decisions [24]. Institutions that are more experienced with implementation may realize this strategy alone is not sufficient for implementation success and have appointed formal implementation leaders to oversee all phases of implementation and provide a cohesive strategy. Dedicated precision medicine or PGx teams have been cited as leading these types of implementations [25, 26]. Another construct that was identified as important but not necessarily different by class included patient needs and resources. Educating patients about their PGx results along with how it may impact response to drug therapy is an important component of PGx implementation. Greater patient knowledge about genetics has been associated with favorable attitudes towards PGx testing [27]. Patient resources such as the ability to pay for testing or insurance coverage for testing will also need to be considered.

These results highlight how priorities may shift as organizations progress through the various phases of implementation. However, some organizations may have different priorities based on their mission, organizational setting, and the populations they serve. Therefore, results may reflect inherent differences in organizational characteristics rather than an evolution through implementation maturity. Here it is worthwhile to consider that BWS latent class analysis may be most useful as a first step in a sequential mixed methods study in which the next step would involve qualitative research to explain and confirm the BWS findings with stakeholders.

Few centers have formalized processes for clinical PGx testing due to several implementation challenges [28, 29]. Our latent class analysis of the BWS ranking of CFIR constructs in centers that have implemented or are planning to implement PGx testing aligns with the results of previous studies that have reported on facilitators and barriers of PGx implementation [17, 28–30]. These studies have previously identified the limited evidence for clinical utility, unclear cost-effectiveness for PGx testing, and limited physician education regarding PGx testing as major barriers limiting PGx adoption. These barriers were identified by providers or physicians, who are important, but not the sole stakeholders in the implementation process. Additionally, reimbursement for PGx testing is rapidly evolving with Medicare and several private insurers now reimbursing PGx testing [31]. While the constructs of evidence strength and quality, cost, and knowledge and beliefs were also identified in our study, we previously published the process these early adopter sites deployed in overcoming these challenges when implementing PGx testing for antidepressants such as creating educational modules for providers about PGx [11].

This study has some limitations. The sample size was small, limiting statistical inference related to differences in priorities by class. Respondents in the study represented US-based organizations participating in the IGNITE network; therefore, the findings reflect priorities of early adopters of PGx testing in the US and the rankings of CFIR constructs may be quite different for subsequent adopters of PGx testing and for implementers outside of the US. Representatives from each of the 17 sites were asked to respond to the survey providing a consensus on prioritization from their site, which may have varied in terms of composition (i.e., implementers, mental health providers, pharmacists, administrators) and could have influenced the survey response. However, processes for implementing PGx testing and factors important for implementation are relevant for centers seeking to begin implementation.

## Conclusion

This study demonstrates the feasibility of applying BWS methodology from the perspectives of the organization to capture the importance of factors for implementing pharmacogenetic testing. Organizations were consistent and ranked patient needs and resources, leadership engagement, knowledge and beliefs about the intervention, evidence strength and quality, and identifying champions as the most important constructs for pharmacogenetic implementation. Experience from early adopters in our study identifies which important constructs and the order in which they should be addressed when designing strategies for the implementation of PGx testing. These rankings were confirmed by the latent class analysis. Future research should evaluate the use of BWS when evaluating implementation strategies for PGx testing.

## Supplementary Information

**Additional file 1.** Electronic survey.

**Additional file 2.** STROBE statement.

**Additional file 3.** Characteristics of sites that had implemented  or were planning to implement pharmacogenetic testing to guide antidepressant therapy.

## Data Availability

The datasets used and/or analyzed during the current study are available from the corresponding author on reasonable request.

## Abbreviations

PGx: Pharmacogenetic
IGNITE: Implementing Genomics in Practice network
CFIR: Consolidated Framework for Implementation Research
BWS: Best-worst scaling
