# Supplementary material for: Best–worst scaling methodology to evaluate constructs of the Consolidated Framework for Implementation Research: application to the implementation of pharmacogenetic testing for antidepressant therapy
Source: Implement Sci Commun. 2022 May 14;3:52. doi: 10.1186/s43058-022-00300-7 (PMC9107643; doi:10.1186/s43058-022-00300-7)
Supplement: Supplementary file 1 — Additional file 1. Electronic survey. [file 43058_2022_300_MOESM1_ESM.pdf]

Resize font: ⊞ | ⊟

Returning?

IGNITE2: Antidepressant Pharmacogenetic Implementation

Informed Consent

Project Title

IGNITE 2: Pharmacogenomics in Practice

You are invited to participate in a research study to survey individuals involved in pharmacogenetic implementation to assess strategies at your institutions for providing genotype-guided therapy related to treatment of depression and other diseases.

Purpose of this Survey

To assess implementation strategies, priorities, challenges encountered, and lessons learned across multiple early adopters of pharmacogenetics in practice for genotype-guided therapies, including selection of antidepressant medications and preemptive testing approaches.

Procedures to be Followed

If you agree to be in this study, you will be asked to complete the survey, which is in the form of a link to a REDCap survey included in this email. Please complete the survey. The survey results will be sent to the study investigators to be aggregated with responses from other institutions.

Duration

The survey should take about 30 minutes to complete.

Risks and Benefits of Participation

The study has minimal risk. The benefits of you participating in the study are being a co-author on a published paper and abstract presented at scientific meetings.

Statement of Confidentiality

The completed survey will be saved in REDCap. Once all the sites have completed and returned the surveys, the data from all sites will be collated in tables, figures, and text and shared in the form of an abstract and/or manuscript for publication.

Privacy Authorization

Only certain people will have the legal right to collect, use and/or give out information collected in this survey and they will protect the privacy and security of these records to the extent the law allows. These people include:

· The study Principal Investigator and research staff associated with this project

· Other professionals at UF that provide study-related procedures

· The Institutional Review Board (IRB)

Voluntary Participation

Participation in this study is voluntary

Whom to contact if you have questions about the study:

Principal Investigator: Larisa H. Cavallari, PharmD, Phone: 352-273-8245

Whom to contact about your rights as a research participation in the study:

IRB02 Office, Box 11225, University of Florida, Gainesville, FL 32611-2250; phone (352) 273-9600.

Institution Name

* must provide value

Institution location (city, state)

* must provide value

Which of the following best describes your institution (check all that apply) ?

* must provide value

- Academic medical center (inpatient/outpatient)
- Nonprofit hospital
- For profit hospital
- Nonprofit ambulatory care clinic
- For profit ambulatory care clinic
- Veteran Affairs (VA) hospital

Stage in antidepressant pharmacogenetic implementation process

* must provide value

- Implemented
- Planning

reset

Note: for the remaining questions, institutions in the planning phase should answer to the extent that the information is known or planned at the current time. If needed, please offer additional comments in the 'other' selection options or 'other comments' at the end of the survey.

Testing approach

* must provide value

- Preemptive
- Reactive
- Both

reset

Who was the 'primary champion' for the initiating implementation efforts for antidepressant pharmacogenetics?

* must provide value

- Pharmacy
- Psychiatry
- Primary care
- Precision medicine/Pharmacogenetics service
- Health informatics
- Nursing
- Psychology
- Other

reset

Who is currently leading the pharmacogenetic implementation effort for antidepressants (check all that apply)

* must provide value

- Pharmacy
- Psychiatry
- Primary care
- Precision medicine/Pharmacogenetics service
- Health informatics
- Nursing
- Psychology
- Other

Who are collaborators in the pharmacogenetic implementation effort for antidepressants (check all that apply)

* must provide value

- Pharmacy
- Psychiatry
- Primary care
- Precision medicine/Pharmacogenetics service
- Health informatics
- Nursing
- Psychology
- Other

Which clinical service lines offer pharmacogenetic testing for antidepressant guidance? (check all that apply)

* must provide value

- Pharmacy
- Psychiatry
- Primary care
- Pediatrics
- Neurology
- Other

Are there specific diagnostic indications required for testing?

* must provide value

- Yes
- No

reset

Are there specific prior treatment utilization characteristics required for testing?

* must provide value

- Yes
- No

reset

Who initiates an order for a pharmacogenetic test for antidepressant guidance? (check all that apply)

* must provide value

- PGx service prescriber
- Psychiatry prescriber
- Primary care prescriber
- Pharmacist
- Other

What is the context of delivery of pharmacogenetic testing for antidepressants?

* must provide value

- Clinical care
- Research
- Clinical care and research

reset

Where is antidepressant pharmacogenetic testing offered at your institution?

* must provide value

- Inpatient
- Outpatient
- Both inpatient and outpatient

reset

Who initiates the order for antidepressant pharmacogenetic testing (check all that apply)?

* must provide value

- Pharmacogenetic service
- Psychiatry
- Primary care
- Other

Who identifies patients for pharmacogenetic testing to guide antidepressant use (check all that apply)?

* must provide value

- Prescriber
- Pharmacist
- Best practice alert
- Patient self-referral
- Other provider

Who provides the pharmacogenetic testing (check all that apply)?

* must provide value

- Hospital/academic clinical lab at your institution
- Hospital/academic clinical lab at another institution
- Commercial lab
- Research lab

What is the test ordering process?

* must provide value

- Prescriber orders commercial test directly through commercial lab
- Prescriber orders commercial test through institutional lab
- Research protocol
- Other

reset

How is testing paid for (check all that apply)?

* must provide value

- Patient
- Insurance/third party billed
- Research
- Other

What type(s) of pharmacogenetic tests are currently ordered at your institution (check all that apply)?
* must provide value

- Single gene tests (e.g. CYP2D6 and CYP2C19 testing run separately)
- Multigene tests (panel)
- Other

What type(s) of method is used to analyze DNA at your institution (check all that apply)?
* must provide value

- Sequencing
- Genotyping
- Unknown

Is there an established institutional workflow around the ordering of tests and return of results?
* must provide value

- Yes
- No

reset

How are results returned to provider (check all that apply)?
* must provide value

- Electronic Medical Record lab result
- Electronic Medical Record consultation note
- Prescriber specific portal for commercial test
- Mail or fax
- Other

Are the pharmacogenetic results discrete data in the electronic health record?
* must provide value

- Yes
- No

reset

Who returns results to the patient (check all that apply)?
* must provide value

- Prescriber
- Pharmacist
- Genetic counselor
- Patient receives results directly from lab (no provider involved)
- Results not returned directly to the patient (e.g. research protocol)
- Other

How are results returned to the patient (check all that apply)?

* must provide value

- Paper copy
- PDF
- Patient specific portal (e.g. for commercial test or EMR access)
- Letter
- Consultation note
- EMR message
- Verbal return of results
- Other

Please list the average turnaround time for results (days)

* must provide value

How are results stored in the EMR? (check all that apply)

* must provide value

- Lab results section
- Allergy list
- Problem list
- PDF upload
- Pharmacogenomics/Pharmacogenetics section
- Other

Which genes are used to guide antidepressant therapy at your institution (check all that apply)?

* must provide value

- CYP2D6
- CYP2C19
- Other

What support is available to the healthcare system to use the available genetic information for other prescribing decisions? (check all that apply)

* must provide value

- PDF report
- Clinical decision support
- Consultation
- None
- Other

Are pharmacogenetic results ordered for antidepressant guidance used for other purposes (e.g. CYP2D6 for codeine guidance, CYP2C19 for clopidogrel, etc) ?

* must provide value

- Yes
- No
- Unclear

reset

Which antidepressants are considered for pharmacogenetic guidance? (check all that apply)

* must provide value

- Citalopram
- Escitalopram
- Sertraline
- Paroxetine
- Fluvoxamine
- Fluoxetine
- Duloxetine
- Venlafaxine
- Vortioxetine
- Tricyclic Antidepressants
- Other

Which factors influence the antidepressants considered for pharmacogenetic guidance (check all that apply)?

* must provide value

- CPIC guidelines
- FDA labeling
- Dutch PGx Working Group Guidelines
- Suggestions or recommendations from the commercial laboratory
- Other

Which age categories are eligible for antidepressant pharmacogenetic guidance at your institution?

* must provide value

- ≥18
- <18
- No age restriction

reset

How many PGx tests for antidepressants have been ordered to date for a patient with an ICD code for depression or currently taking an antidepressant medication at your institution?

How many PGx tests for antidepressants were ordered within the past year?

Other comments

Expand

Submit

Save & Return Later

Survey 2

You are invited to participate in a research study to survey individuals involved in implementation of pharmacogenetics to guide antidepressant pharmacotherapy.

Purpose of this Survey

Barriers to implementation can arise at several levels of healthcare delivery: the patient level, provider team level, the organizational level, or policy level. **The goals of this survey are to**:

1. Identify which factors were most important when implementing PGx for antidepressants at your institution;
2. Identify outcomes you measured to assess the success of the implementation.
3. Describe the implementation strategies that were most effective during your implementation.

We will ask you a series of questions to understand the importance of these factors. If you have not implemented PGx testing or are in the planning stages of implementation, please indicate those factors that you perceive will be important. The survey should be answered using the health system perspective. It is recommended that you discuss the survey with other member of your PGx implementation team (e.g. Precision Medicine administrator, psychiatrist, pharmacist) before completing the survey.

Procedures to be Followed

If you agree to be in this study, you will be asked to complete the survey, which is in the form of a link to a Sawtooth survey included in this email. The link is unique to you; please do not forward to other individuals. Please complete the survey. The survey results will be sent to the study investigators to be aggregated with responses from other institutions.

Duration

The survey should take about 45-60 minutes to complete. Your responses will be saved in the event you cannot complete the survey in one sitting and need to return to it at a later time.

Risks and Benefits of Participation

The study has minimal risk. The benefits of you participating in the study are being a co-author on a published paper and abstract presented at scientific meetings.

Statement of Confidentiality

The completed survey will be saved in Sawtooth. Once all the sites have completed and returned the surveys, the data from all sites will be collated in tables, figures, and text and shared in the form of an abstract and/or manuscript for publication.

Privacy Authorization

Only certain people will have the legal right to collect, use and/or give out information collected in this survey and they will protect the privacy and security of these records to the extent the law allows. These people include:

· The study Principal Investigator and research staff associated with this project
· Other professionals at UF that provide study-related procedures
· The Institutional Review Board (IRB)

Voluntary Participation

Participation in this study is voluntary

Whom to contact if you have questions about the study:

Principal Investigator: Larisa H. Cavallari, PharmD, Phone: 352-273-8245

Whom to contact about your rights as a research participation in the study:

IRB02 Office, Box 11225, University of Florida, Gainesville, FL 32611-2250; phone (352) 273-9600.

Directions for Survey Completion:

Please fill out the following survey to best of your abilities. If your institution is has not yet implemented pharmacogenetic testing for mental health conditions or are in the planning stages of implementation, please indicate those factors that you perceive will be important. The survey should be answered using the health system perspective.

By proceeding with the survey, you are indicating consent to participating in the study.

If you have any questions or require clarification, please feel free to contact Sony Tuteja, PharmD, MS (sonyt@pennmedicine.upenn.edu)

Implementation Science Survey

1. Name of completer: ______________________
2. Email: _________________________
3. Site name: ________________________
4. Role on team:
    a. Precision Medicine
    b. Pharmacy/Pharmacology
    c. Psychiatry
    d. Pathology/Lab Medicine
    e. IT EHR team
    f. other___________
5. List others team members providing input on survey along with their role:
    a. ____
    b. ____
    c. ____

[This section will be the discrete choice experiments]

The first section will ask you to identify the importance of factors external to your organization such as the economic, political, and social context surrounding your healthcare system's infrastructure. It is not necessary to remember what you selected on the previous screen, just select the most and least important factor on the current screen.

1. Patient needs and resources that are tied to social determinants of health (e.g. patients network, housing, poverty, food)

2. Patient needs and resources tied to individual, or bio-psychosocial factors (e.g. age, sex/gender, language, literacy, insurance, clinical history, family history, genetics, self-reported health, medication adherence)

3. The degree to which your organization is networked with other organizations

4. Peer pressure (competitive pressure to implement PGx testing for antidepressants because other competing organizations have already implemented)

5. External policy and incentive to spread PGx testing for antidepressants including policy and governmental regulations guidelines, pay-for-performance, or public or reporting.

The next section will ask you to identify the importance of factors internal to your organization such as the structural, political, and cultural contexts within your institution.

6. Social structure characteristics (e.g. the age and size of your organization)

7. The nature and quality of social networks and the nature of formal and informal communications within your organization

8. Cultural norms and values of your organization

9. Implementation climate including the capacity for change and shared receptivity of involved individuals to PGx testing for antidepressants

10. The degree to which stakeholders perceive the current situation as intolerable or needing change

11. The degree of compatibility between meaning and values attached to PGx testing for antidepressants by involved individuals and how those align with individuals' own values, and perceived risks and needs, and how testing fits with existing workflows and systems

12. Individuals' shared perception of the importance of the implementation within your organization

13. Organizational incentives and rewards (Extrinsic incentives such as performance reviews, promotions, raises in salary and increased stature)

14. Goals and feedback (The degree to which goals are clearly communicated, acted upon, and fed back to staff and alignment of that feedback with goals)

15. A learning climate in which: leaders express their own fallibility and need for team members' assistance and input; and team members feel that they are essential, valued, and knowledgeable partners in the change process

16. Tangible and immediate indicators of organizational commitment and readiness to its decision to implement PGx testing for antidepressants.

17. Leadership engagement (Commitment, involvement, and accountability of leaders and managers with the implementation)

18. Available resources (The level of resources dedicated for implementation and on-going operations including money, training, education, physical space, and time)

19. Access to knowledge and information (Ease of access to digestible information and knowledge about PGx testing for antidepressants and how to incorporate it into work tasks.)

The next section will ask you to identify the importance of factors related to the behavioral constructs of clinicians involved with deploying PGx testing to guide antidepressant use and/or its implementation.

20. Clinician's knowledge and beliefs about PGx testing for antidepressants

21. Clinicians' belief in their own capabilities to execute courses of action to achieve implementation goals

22. Individual stage of change (Characterization of the phase an individual is in, as he or she progresses toward skilled and sustained use of PGx testing for antidepressants)

23. Individual Identification with Organization (how individuals perceive your organization and their relationship and degree of commitment with the organization)

24. Other personal attributes (other personal traits such as intellectual ability, motivation, values, competence, capacity, and learning style)

The next section will ask you to identify the importance of factors related to the characteristics of the PGx testing.

25. Intervention source (Perception of key stakeholders about whether PGx testing is externally or internally developed)

26. Evidence Strength & Quality (Stakeholders' perceptions of the quality and validity of evidence supporting PGx for antidepressants management)

27. Stakeholders' perception of the advantage of implementing PGx to guide antidepressants versus an alternative solution

28. The degree to which antidepressant PGx can be adapted, tailored, refined, or reinvented to meet local needs

29. The ability to test antidepressant PGx on a small scale in the organization, and to be able to reverse course (undo implementation) if warranted

30. Perceived difficulty of implementation, reflected by duration, scope, disruptiveness, and number of steps required to implement

31. Perceived excellence in how antidepressant PGx is presented and assembled

32. Costs of PGx testing and costs associated with implementing that intervention including investment, supply, and opportunity costs

33. The ability of the healthcare system to educate individuals receiving care, families, clinicians.

The next section will ask you to identify the importance of factors related to the essential activities of the implementation process.

34. Planning (The degree to which methods for implementing PGx testing for antidepressants are developed in advance and the quality of those methods.)

35. Engaging (Attracting and involving appropriate individuals in the implementation and use of PGx testing for antidepressants through a combined strategy of marketing, education, and training)

36. Opinion leaders (Individuals in your organization who have formal or informal influence on the attitudes and beliefs of their colleagues with respect to implementing the intervention)

37. Formally appointed internal implementation leaders (Individuals from within your organization who have been formally appointed with responsibility for implementing an intervention as coordinator, project manager, or team leader)

38. Champions (Individuals who dedicate themselves to support or market PGx testing and help to overcome indifference or resistance to the intervention)

39. External change agents (Individuals who are affiliated with an outside entity who formally influence or facilitate PGx decisions in a desirable direction)

40. Executing (Carrying out or accomplishing the implementation according to plan)

41. Reflecting and evaluating (Quantitative and qualitative feedback about the progress and quality of implementation accompanied with regular personal and team debriefing about progress and experience)

42. What outcomes are you/will be measuring as part of your implementation? (check all that apply)

1 □ Feasibility- the extent to which the PGx testing can be successfully used or carried out within a setting

2 □ Fidelity- degree to which PGx testing was implemented as it was intended

3 □ Penetration- integration of PGx testing within a service setting

4 □ Acceptability- degree to which PGx testing is agreeable, palatable or satisfactory

5 □ Sustainability- the extent to which PGx testing is maintained

6 □ Adoption – intention, initial decision, or action to try or employ PGx testing

7 □ Implementation Cost- cost impact of a PGx implementation effort

8 □ Efficiency- avoiding waste, including waste of equipment, supplies, ideas, and energy

9 □ Safety- avoiding harm to patients from the care that is intended to help them

10 □ Effectiveness- providing services based on scientific knowledge to all who could benefit and refraining from providing services to those not likely to benefit

11 □ Equity- providing care that does not vary in quality because of personal characteristics such as gender, ethnicity, geographic location and socioeconomic status

12 □ Patient-centeredness- providing care that is respectful of and responsive to individual patient preferences, needs, and values and ensuring that patient values guide all clinical decisions

13 □Timeliness- Reducing waits and sometimes harmful delays for both those who receive and those who give care.

14 □Satisfaction- patient satisfaction

15 □ Function- quality of life

16 □ Symptomatology- patient reported symptoms relating to disease or treatments

17 □ Impact on health and social policy (healthcare, educational, public health, environmental, industry, laws regulation)

An implementation strategy is defined as methods or techniques used to enhance the adoption, implementation, and sustainability of a clinical program or practice.

43. Which of the following strategies have you employed or are planning on employing when implementing PGx testing for antidepressants at your institution? (check all that apply)

1 □ Identify barriers for implementation

2 □ Conduct a local needs assessment by collecting and analyzing data related to PGx implementation.

3 □ Develop a formal implementation blueprint

4 □ Obtain and use patient and family feedback

5 □ Provide centralized PGx consultation and technical assistance

6 □ Identify and prepare PGx champions (Clinicians and/or other staff who dedicate themselves to leading, supporting and marketing the PGx testing effort to overcome indifference or resistance)

7 □ Identify early adopters (Those charged to begin implementing the PGx testing in their practice and those that are already applying PGx testing so that other can learn from and even be inspired by their experiences)

8 □ Use advisory boards and workgroups

9 □ Develop educational materials for patients

10 □ Develop educational materials for providers

11 □ Develop educational materials for pharmacists

12 □ Conduct ongoing training

13 □ Facilitate relay of PGx test results within the electronic health record to providers

14 □ Create or change infrastructure in the electronic health record

15 □Other: __________________________

44. List the top 3 strategies that were the most effective in implementing PGx testing for antidepressants and why: (open-ended)

45. How are you funding the implementation? (check all that apply)
    a. External grants (NIH, foundation)
    b. Internal Health system or University funding
    c. Philanthropy
    d. Industry partnership
    e. Clinical revenue
    f. Other _______________________

46. Please provide any additional comments you wish to share regarding your PGx implementation. (open ended)
