# Supplementary material for: Best–worst scaling methodology to evaluate constructs of the Consolidated Framework for Implementation Research: application to the implementation of pharmacogenetic testing for antidepressant therapy
Source: Implement Sci Commun. 2022 May 14;3:52. doi: 10.1186/s43058-022-00300-7 (PMC9107643; doi:10.1186/s43058-022-00300-7)
Supplement: Supplementary file 3 — Additional file 3. Characteristics of sites that had implemented or were planning to implement pharmacogenetic testing to guide antidepressant therapy. [file 43058_2022_300_MOESM3_ESM.pdf]

Additional File 3. Characteristics of sites that had implemented or were planning to implement pharmacogenetic testing to guide antidepressant therapy

Institution Type	Institution Name	Antidepressant Launch Year	Testing Approach	Testing Setting	Model	Services Offering Testing
Implemented						
Academic Medical Center	Cincinnati Children's Hospital Medical Center	2004	Reactive and preemptive	Inpatient and outpatient	Clinical care	Psychiatry<br>Pediatrics
Academic Medical Center	Indiana University	2016	Reactive	Inpatient and outpatient	Clinical care	Psychiatry
Academic Medical Center	Michigan Medicine	2018	Reactive	Outpatient	Clinical care	Psychiatry<br>Primary Care<br>Pediatrics<br>Neurology
Academic Medical Center	Moffitt Cancer Center	2018	Preemptive	Inpatient and outpatient	Research	Psychiatry
Academic Medical Center	University of Alabama at Birmingham	2018	Reactive	Outpatient	Clinical care	Psychiatry
Academic Medical Center	University of Colorado/UCHealth	2019	Preemptive	Inpatient and outpatient	Clinical care and research	All Services
Academic Medical Center	University of Florida Health	2016	Reactive and preemptive	Outpatient	Clinical care and research	All Services
Academic Medical Center	Vanderbilt University Medical Center	2010	Reactive and preemptive	Inpatient and outpatient	Clinical care and research	Psychiatry<br>Primary Care<br>Pediatrics<br>Neurology
Nonprofit Hospital, Nonprofit Ambulatory Care	Intermountain Healthcare Precision Genomics	2017	Reactive and preemptive	Inpatient and outpatient	Clinical care	All Services
Nonprofit Hospital, Nonprofit Ambulatory Care Academic Medical Center	MedStar Health	2019	Reactive and preemptive	Outpatient	Clinical care and research	All Services

Nonprofit Hospital, Nonprofit Ambulatory Care	Nemours Children Health	2020	Reactive	Inpatient and outpatient	Clinical care	Pharmacy<br>Psychiatry<br>Primary Care<br>Pediatrics<br>Neurology
Nonprofit Hospital	Sanford Health	2015	Reactive and preemptive	Inpatient and outpatient	Clinical care	Psychiatry<br>Primary Care<br>Pediatrics
Veteran Affairs Hospital	Durham VA Medical Center	2019	Reactive and preemptive	Inpatient and outpatient	Clinical care	All services
Planning						
Academic Medical Center	University of North Carolina Medical Center	2021	Preemptive	Outpatient	Research	TBD
Academic Medical Center	University of Pennsylvania	2022	Reactive	Outpatient	Clinical care	Psychiatry<br>Medical Genetics
Academic Medical Center	University of Pittsburgh/UPMC	2021	Reactive and preemptive	Inpatient and outpatient	Clinical care and research	Pharmacy<br>Primary Care
Nonprofit Hospital	M Health Fairview	2021	Reactive and preemptive	Inpatient and outpatient	Clinical care	Pharmacy<br>Psychiatry<br>Primary Care
